# Musical Breaks—Live Music in a Hemodialysis Setting—A Qualitative Study on Patient, Nurse, and Musician Perspectives

**DOI:** 10.3390/healthcare10091637

**Published:** 2022-08-27

**Authors:** Margrethe Langer Bro, Jeanette Finderup, Rineke Smilde, Bibi Gram, Pia Dreyer

**Affiliations:** 1 The Royal Academy of Music, 8000 Aarhus, Denmark; 2 The Danish National Academy of Music, 6700 Esbjerg, Denmark; 3 Department of Renal Medicine, Aarhus University Hospital, 8000 Aarhus, Denmark; 4 Department of Clinical Medicine, Aarhus University, 8000 Aarhus, Denmark; 5 ResCenPI—Research Centre for Patient Involvement, Aarhus University, Central Denmark Region, 8000 Aarhus, Denmark; 6 Centre of Applied Research and Innovation ‘Art and Society’ of the Hanze University of Applied Sciences, 9747 Groningen, The Netherlands; 7 Department of Lifelong Learning in Music, University of Music and Performing Arts, 1030 Vienna, Austria; 8 Research Unit of Health Science, Hospital of South West Jutland, University Hospital of Southern Denmark, 6700 Esbjerg, Denmark; 9 Department of Regional Health Research, University of Southern Denmark, 5000 Odense, Denmark; 10 Department of Intensive Care, Aarhus University Hospital, 8000 Aarhus, Denmark; 11 Department of Public Health, Section of Nursing, University of Aarhus, 8000 Aarhus, Denmark; 12 Department of Global Public Health and Primary Care, University of Bergen, 5007 Bergen, Norway

**Keywords:** hemodialysis, end-stage kidney disease, music intervention, qualitative research, musician

## Abstract

The aim of this hermeneutic-phenomenological study was to explore the perspectives of 12 patients, 17 nurses, and 4 musicians on patient-tailored live music interventions in a hemodialysis setting. Twenty-six semi-structured interviews were collected—17 with patients, 9 with nurses. Furthermore, 18 moderate participation observations, whilst 1 semi-structured group interview with 3 nurses and 3 musicians, and 13 reflective journals from musicians were collected. Within the analysis—based on Ricoeur’s theory of interpretation—two overall themes emerged: (1) the inner space and (2) the participating space, followed by five subthemes: (1a) Entering a calm and enjoyable pause bubble; (1b) Resting in a thought-free state of mind; (1c) Traveling in the past and catching the moment through heartfelt music; (2a) Bringing positive changes into life; (2b) The artistic quality mediating a magnificent and beautiful experience. We found that patient-tailored live music was a meaningful break, influencing mental and physical well-being, time perception, community, work environment, and artistic approach. The artistic quality of the music was essential—together with the musicians’ social awareness, empathy, and ability to interact with the patients—in creating meaningful moments for patients and staff. Overall, the music interventions were a welcome change in a predictable world of stressful routines and repetitive treatments.

## 1. Introduction

For decades, music-based interventions have been used in different healthcare settings. A recent report published by the World Health Organization identified over 3000 studies showing a major role for the arts in the prevention of ill health, promotion of health, and management and treatment of illness throughout life [1]. However, less is known about the role of the arts in a hemodialysis (HD) setting. Therefore, this study set out to investigate the potential of music-based interventions in this environment.

Undergoing HD treatment has a profound impact on patients’ quality of life and physical and mental health, and, for many, is something they will have to do regularly for the rest of their life [2,3,4]. “Debilitation and exhausting burden” and “restricted life participation” were among the themes identified in a systematic review and thematic analysis of patients in HD treatment, based on qualitative data from 65 studies (*n* = 1731) [5]. In one study where 12 older (75+ years) patients receiving HD were asked about what mattered most in life to them, the two overarching themes that emerged were “having physical well-being” and “social support” [6]. In another study, the patients associated their HD treatment with existential boredom and killing and wasting time, and the authors recommended that nurses should spend more time interacting and communicating with the patients, as well as the provision of additional recourses and facilities to help patients pass the time during treatment [7]. Additionally, developing tools to reduce boredom and facilitate community and engagement in meaningful tasks was recommended in an interview study that involved 11 patients commencing HD. It is possible that developing such tools or initiatives could help patients on HD maintain a sense of continuity in their self-perception of identity [8].

The literature shows that nine out of 10 randomized controlled trials applying music interventions during HD found significant effects on anxiety, pain, depression, stress, sleep, and relaxation compared to a control group [9,10,11,12,13,14,15,16,17,18]. These studies were overwhelmingly conducted by nursing teams, and the proposed intervention was based on listening to music, either recorded [11,12,13,14,15,16,17,18] or live music played by nurses during HD sessions [9,10]. A recent systematic review and meta-analysis found that music interventions effectively reduced pain symptoms in patients on HD [19]. To date, no studies have explored to what extent patient-tailored live music interventions performed by highly skilled professional musicians can improve well-being and create a sense of coherence and meaningfulness among patients, staff, and musicians within an HD setting. 

Internationally, partnerships have emerged in recent years to examine a triangle approach to patient, nurse, and musician perspectives. One example is the Dutch project Meaningful Music in Health Care [20,21], which uses person-centered live music on surgery wards performed by highly skilled musicians for the wellbeing of patients, as a catalyst for compassionate patient relationships, as well as a tool to improve a hectic work environment for healthcare professionals [21,22,23]. In that context, cooperation between nurses and musicians is the key to success. From the healthcare professional’s perspective, the Dutch research group has shown that music interventions in a surgery ward create a new conscious and in-depth understanding of the patient’s situation, promoting a cultural shift from a task-centered approach towards a relationship-focused, person-centered approach. These experiences created emotional resonance, enabling nurses to “look through the eyes of others”, which was crucial to their perception of the value of music, as well as similarities between their own and the musicians’ approaches to person-centered care and patient-tailored music, respectively [23].

It has been found that nurses in an HD setting, witnessing patients with a complex and chronic illness, experience moderate to high levels of burn-out and that new efforts are needed to ameliorate this stressful work environment [24,25]. It has been proved that a healthy work environment has a profound effect on the well-being of nurses working in an HD unit. Their well-being is a prerequisite for the provision of high-quality healthcare services to patients [24].

For musicians, high-quality music standards combined with empathy and social awareness are prerequisites for patient-tailored music performances, using more intuitive artistry to target new audiences in the healthcare setting [21,23,26,27,28]. This practice requires increased artistic awareness—a dialogue between the inner and the outer, between the artist and their sensibility to social needs [26]. According to Peter Renshaw, “it is important for health care musicians to always remain true to their own artistic identity and authenticity,” using their skills and commitment to create joy and improve the quality of life for patients in hospitals and care centers through specially designed and personalized live performances [26,27,28]. In contrast to music therapy, where an individual’s goal of achieving a “therapeutic relationship” is pivotal [29], this practice does not have an intended treatment component [30]. The primary focus is on the music itself, not as a means “to achieve something;” that is, the music is here defined as having a unique aesthetic value in itself, conveyed through emphatic, sensitive, and situational music practice [31]. No previous studies had used a triangle focus to investigate what music interventions in HD mean for patients, nurses, and musicians. Therefore, based on the background outlined above, the aim of this study was to explore the perspectives of patients, nurses, and musicians on patient-tailored live music interventions in an HD setting.

## 2. Materials and Methods

### 2.1. Study Design

This phenomenological-hermeneutic qualitative study was an integrated part of a randomized controlled pilot trial where patients undergoing HD treatment were randomized to receive one patient-tailored live music intervention weekly for a period of six weeks as an add-on to treatment as usual. Qualitative data were collected using various methods, including moderate participation observations, semi-structured interviews with individual patients and staff, a semi-structured group interview with both staff and musicians, and reflective journals from musicians. This study is reported according to the consolidated criteria for reporting qualitative research (COREQ) [32].

Data from the randomized controlled pilot trial will be published separately, with the aim of evaluating the feasibility of the patient-tailored live music intervention and its possible effectiveness on fatigue, depression, anxiety, relaxation, treatment satisfaction, well-being, and musical experience among patients undergoing HD, as well as levels of work engagement among nurses in the clinic.

### 2.2. Participants

We recruited 24 participants on HD treatment at one satellite unit at a regional hospital in Denmark. Eligible patients were aged 18 or over. We excluded patients with mental illness or deafness, and those not able to understand or speak Danish. The patients were divided into two groups: one group (*n* = 12) received HD treatment Monday, Wednesday, and Friday, and the other group received HD treatment Tuesday, Thursday, and Saturday. The two groups were randomized (1:1) either to receive the intervention one day a week for six weeks plus usual care or only usual care. This qualitative study only covers the 12 patients who received the intervention. In addition, 17 healthcare professionals (nurses) in the HD unit and 4 professional musicians participated in the study. The participants were identified as follows: IP1–12 (interview patients), IN1–17 (interview nurses), and IM1–4 (interview musicians). Those who took part in the group interview were identified as GN1, GN2, and GN17 (group nurses) and GM1–3 (group musicians), and the reflective journals as RF1–4 (musicians).

### 2.3. Interventions

The study involved 30 min patient-tailored music interventions, for three groups of four patients each, during HD treatment, provided once a week over a period of six weeks. The musicians worked in shifts to provide the interventions, performing music that was calming and uplifting, with different styles, tempos, and musical expressions, to meet the patients’ individual needs and situations.

Two months before the intervention period, four professional musicians—one guitar duo and two solo guitarists (all acoustic instruments) from the Danish organization Live Music in Public Schools—received a three-day theoretical and performative program to develop and ensure a high musical standard and to give them the skills to tailor the music to individual patients. The musicians were also informed about the life circumstances of patients on HD treatment and the hospital routines, setting, and acoustics. Two weeks before the intervention period, they had a conversation with the staff about cooperation and logistical challenges and tested the acoustics of the ward for themselves.

### 2.4. Data Collection

An overview of the data collection is presented in Table 1. Eighteen moderate participation observations, seventeen semi-structured interviews with patients, nine semi-structured interviews with nurses, one semi-structured group interview, and thirteen reflective journals from the musicians were collected. Eight patients were interviewed once, while four were interviewed repeatedly, using the original interview guide (IP1 = 2, IP4 = 2, IP3 = 2, IP10 = 3) to capture their experiences of the variety of the music interventions, and possibly the diversity of the musicians’ approaches, musical format, and content. An independent, skilled student assistant collected the moderate participation observations, as well as conducting and transcribing the semi-structured interviews with both patients and nurses. Additionally, three of the four musicians and three of the seventeen nurses participated in a semi-structured group interview after the intervention period.

Moderate participation observations: These observations were used to assess the non-verbal environment in the treatment rooms by focusing on behavior, facial expressions, body language, moods, and actions among the patients, nurses, and musicians during the music interventions. The method of moderate participation observations—developed by Spradley—allows the observer to maintain “a balance of being an insider and an outsider, between participation and observation,” and thereby underspin and enhance the patient and nurse interviews [33]. An observation guide was used for the observations, and the observation data were analyzed together with the interview data [33].

Semi-structured interviews (patients and staff): the interviews were conducted as a casual conversation in which questions emerged based on the informants’ comments and the intervention that had just been observed. The format was based on a semi-structured interview guide, but was flexible according to the observations, and the interviewees were free to elaborate on their answers [33]. The aim of these interviews was to generate data about specified topics—e.g., perceptions of the music interventions—to obtain in-depth knowledge about the patients’ and nurses’ experiences of the music. Each of the interviews (3–5 min) was conducted (and recorded) immediately after a music performance and then transcribed [33].

Semi-structured group interview (staff and musicians): One month after the intervention period, a semi-structured group interview was conducted by the primary investigator and an experienced and independent qualitative observer in order to analyze and interpret the staff’s and musicians’ experiences of participating in the study, and identify any potential cooperation among musicians and staff [34]. An overall group interview guide was developed, inspired by an ongoing European project—MiMiC [20]—where professional musicians performed in healthcare contexts with the general goal of developing mutual awareness of the potential of patient-tailored live music to benefit patients, nurses, and musicians. Therefore, these interviews aimed to draw out insights and best practices regarding the interprofessional collaboration of nurses and musicians [20].

Reflective journals (musicians): After every music session, each musician wrote an entry in a reflective journal, based on the reflection-on-action and reflection-in-action theories developed by Schön [35]. The systematic use of reflective journals is recommended for musicians in such situations to establish a connection between their artistic, personal, and professional awareness of place and identity [35]. This method serves to break habitual ways of thinking and foster a more critical and creative approach to learning. Potentially, this critical reflection may transform a musician’s perception of musicianship into a profound and sensuous artistic and social understanding [28].

### 2.5. Data Analyses

Data were analyzed using the software program NVivo [36]. The analysis was based on the phenomenological-hermeneutic tradition, using a method inspired by the French philosopher Paul Ricoeur, developed by Dreyer and Petersen [37]. Ricoeur argued that phenomenology and hermeneutics are co-dependent and that by combining these approaches, a deeper understanding of the lived experience can be achieved [38]. The method splits structural analysis into three levels: (1) naïve reading; (2) structural analysis; and (3) critical interpretation [37]. The first step involves an open-minded, intuitive, and naïve reading of the text, focusing on which elements touch the interpreter. In step two, the interpreter re-reads the text in order to uncover the structure and the underlying dependencies within it using a dialectical analysis process exploring the textual content, meaning, and emerging themes. In step three, the critical interpretation uses a dialectic process to move from an understanding to an explanation of the text in discussion with relevant theories and research literature [34]. This analytical approach transforms surface interpretation into an in-depth interpretation, thereby developing subjective statements leading to general themes. These themes thus form the basis for a deeper understanding of “being-in-the-world”; in this case, as a patient on HD, a healthcare professional, or a musician in an HD unit [37,39].

## 3. Results

### 3.1. Participant Characteristics

Participant characteristics are presented in Table 2. There were wide ranges in the categories of age, number of months on dialysis, and the number of years working in the dialysis clinic or as a professional musician, respectively. Furthermore, the gender distribution was skewed, except in the patient group.

### 3.2. Musical Content

The classical duo and solo musician performed a wide range of original and transcribed music for classical guitar, such as Tarrega, Vivaldi, Sor, Walton, Bach, and Albeniz, to film music by Stanley Meyers and arrangements of well-known Danish songs by, for instance, Carl Nielsen. The contemporary guitarist performed a mix of well-known Danish songs, film music, and arrangements of popular songs by the Beatles, Eric Clapton, and Carlos Jobim, combined with rhythmical improvisations in bossa nova and blues styles.

### 3.3. Summary of Themes

**Figure 1:**
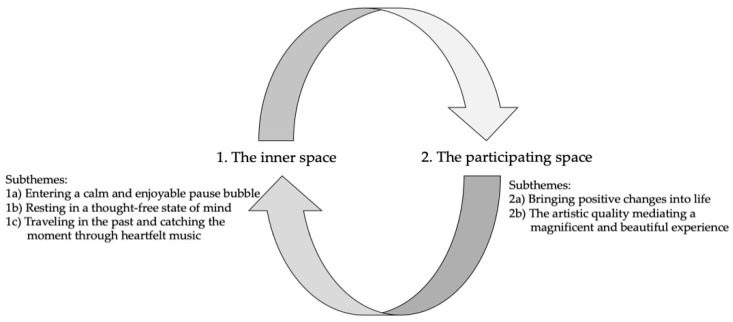
Overview of themes and subthemes identified among patients, nurses, and musicians during HD.

We identified two main themes: (1) the inner space and (2) the participating space, followed by five subthemes among the patients, nurses, and musicians: (1a) entering a calm and enjoyable pause bubble; (1b) resting in a thought-free state of mind; (1c) traveling in the past and catching the moment through heartfelt music; (2a) bringing positive changes into life; and (2b) the artistic quality mediating a magnificent and beautiful experience (Figure 1).

The two main themes created a musical break—a musical space—for patients and nurses in different ways. The inner space gave patients and nurses a moment to relax and let go of negative thoughts, and the participating space provided a source of compassion, common understanding, and meaningfulness among patients, nurses, and musicians (Table 3).

### 3.4. Theme 1: The Inner Space

#### 3.4.1. Subtheme 1a: Entering a Calm and Enjoyable Pause Bubble

According to the patient participants, live music being played in the treatment room gave them an amazing moment of joy and the feeling that this experience was good for both their physical and mental well-being. The music gave patients a warm feeling and calmed them, allowing them to feel more comfortable and relaxed in their body. Patients gave the following observations: “The music was relaxing. It goes straight in. So, it’s really nice.” (IP11); “I’m kind of a little more relaxed in my body. I just fall down a little bit more.” (IP6); “You get better, fit.” (IP1); “Really soothing” (IP10); “A warm feeling.” (IP1). 

The patients also described the music as beautiful, distracting, and a refreshing change that transformed negative feelings into positive ones, such as happiness and joy. One patient commented: “It was just so beautiful. So... Then the thoughts come a little that way, yes.” (IP1). Furthermore, the nurses observed physical changes among the patients, in their facial expressions, body positions, and mental attitudes. For instance, one physical effect of the music interventions was that they reported that the patients would calm down and be able to sleep.

Nurses also emphasized mental calm and positive effects on the mind, making the following remarks: “I think that this gives something good to the mind.” (IN1); “It gives a fantastic calmness.” (IN2); “I just love it.” (IN5). Despite a noisy environment, with alarms from the machines, during the music it was possible to relax, empty one’s mind, let go of worries and bad energy, and enter a quiet and enjoyable bubble where they could de-stress and enjoy the moment. One nurse described it as “a pause bubble... It was just getting out of stress, and just getting down and breathing.” (IN10). This pause bubble helped them to de-stress because “music is all about letting go—letting go of worries and bad energy.” (GN2).

When the musicians played in the treatment rooms, they noticed that the patients seemed uplifted, happy, and grateful for the music. This made the musicians enjoy performing in the HD setting, although it was a new way of performing compared to their primary experience of traditional concerts. The musicians observed that feedback from the patients varied, from eye contact, verbal communication, and smiles, to carefully listening at a distance with their eyes closed. Some patients applauded after the music as if they were attending an ordinary concert, and the musicians sensed that these patients had forgotten that they were in the HD unit: “A few patients sat up in their beds and listened carefully and followed as if they were at a normal concert. Here I felt a clear sign that the patient forgot all about the daily treatment, machines, etc. and was entertained in the same way as at a concert outside the hospital.” (RJ4). Others tapped their feet or shed a tear during the music: “I had eye contact with many of the patients, as well as verbal communication, smiles, and happy faces, while other patients lay down with their eyes closed.” (RJ1).

#### 3.4.2. Subtheme 1b: Resting in a Thought-Free State of Mind

Another experience the patient participants reported was the feeling of being sur-rounded by music, of going into an inner space, an inner echo, of being allowed to be oneself on one’s own terms. The music had brought them into a thought-free state of mind, catching the moment and leaving the world outside. The patients described the feeling in different ways but generally emphasized their own inner space: “One becomes completely surrounded by it.” (IP4); “I was entering my own space.” (IP1); ”It is happening in the world and in the moment, at the same time.” (IP10). It seemed that each patient used the musical experience in their own personal way.

Some of the patients noted that the musical moment gave them a different perception of time. They experienced time as passing faster, which made a difference to these patients undergoing 4–5 h of treatment three times every week, every year, for some, for the rest of their life: “When they [the musicians] have been here, I think it’s nice, because then suddenly 20 min and a half hour or so has passed by. Without really noticing it. So, it’s nice that it [the musical experience] makes time go faster, in a way.” (IP5).

By actively choosing to take a break from work, several nurses felt an impact on their pulse rate and that they could catch their breath. It seemed that going into an inner space helped them let go of stress and work pressure: “Then I chose to prioritize being al-lowed to sit and enjoy it, half the time, I did not have to work at the same time. Well, it’s simply, you... your heart rate goes all the way down and you just get your head emptied and that gives such a break.” (IN10). However, some nurses experienced a tension between being at work and being responsible for the patients and at the same time devoting their attention to the music. They found it distracting and worried that they might overlook important signals from the patients, with serious consequences. One nurse described it in the following way: “As a nurse, you cannot completely indulge in the music because you sit and watch the machines and patients.” (IN17), whereas others stated that they were able to accommodate both the musical experience and their work during the limited 30 min period when the music was played.

#### 3.4.3. Subtheme 1c: Traveling in the Past and Catching the Moment through Heartfelt Music

The musical repertoire was carefully selected, with a mix of well-known pieces in different tempos, styles, and arrangements, and less familiar pieces that were suitable for the setting, taking into account musical structure, harmony, length, and complexity vs. simplicity. To meet the patients´ preferences and situations, different musical expressions were chosen, including romantic, quiet, beautiful, calming, subdued, sad, and happy music: “In this setting, I think that a mix of very subdued, beautiful, romantic, happy, and sad music is appropriate.” (GM3).

The musicians underlined the importance of performing music that they loved and were closely associated with: “The music had to be chosen and played with your heart.” (RJ1).

One of the musicians described it in the following way: “Music is more than just satisfying people. Music should also be used to tease people’s curiosity, enabling openness to something new, I think. So, there are more elements to it.” (GM3).

When the musicians performed well-known pieces, it prompted positive memories: “like traveling in the past,” as one patient noted (IP1), whereas unfamiliar classical guitar pieces brought to mind soothing atmospheres and images of the sea and calming waves: “When it was so quiet and so calm, I thought of the sea. The waves that came rushing just as quietly and so calm, that is. You can imagine some pictures.” (IP11).

Beyond the repertoire, it seemed that the patients´ and staff’s experiences of the music depended on the musician’s presentation and the music’s intensity. One patient highlighted the connection between the quality of the listening experience and how the music was introduced: “I had a bit of dialogue with him. We talked a little bit about it. I think that´s good. At least a change from everyday life here.” (IP7). When the guitar duo decided to play unfamiliar pieces, they made an effort to introduce the music by telling personal anecdotes to create imagery that would help the patients immerse themselves more easily in the music. In contrast, a solo female classical guitarist performed in a constant musical flow which she did not break with verbal communication. Slow, calming, and soothing music made the patients close their eyes and dream away. When the music was performed rhythmically in an energized, up-tempo, and lively way, it encouraged the patients and staff to move: “It gives me either energy or peace.” (IN13). Even though they were not dancers, and they did not describe their activity as dancing, the rhythm made them feel like moving. Patients described it in the following way: “If it´s up-tempo, then you go into it.” (IP11); “Something spontaneous just happens... It’s not the same as dancing. I’m not such a big dancer. But something happens with that rhythm. I feel like moving to the beat of the music.” (IP7).

### 3.5. Theme 2: The Participating Space

#### 3.5.1. Subtheme 2a: Bringing Positive Changes into Life

The semi-structured interviews with the nurses revealed that experiencing the music encouraged the patients to show other sides of themselves, for instance, talking about a family member who was a musician or their own love of a particular Danish composer. Possibly, the music interventions created new connections between the nurses and patients, enabling the nurses to better see the person rather than just the patient, and to direct the conversation towards new shared and positive experiences in the clinic. Furthermore, one patient expressed sympathy and understanding for the staff’s work situation and highlighted the potential benefits of the music for the staff: “It must be a nice change in their lives, with all the mechanics [HD equipment]. With all that they must do [for patients on HD], that must be really nice.” (IP10).

One of the musicians noted that music might act as a “breathing space giving the nurses new energy when they felt sorry for their patients and powerless to be able to improve their situation through their nursing”. This was supported by a nurse saying that the music interventions made her happy and professionally proud when she noticed how the initiative benefitted this group of patients. Another nurse stated that the music provided a special, encouraging, and life-affirming moment for one male patient, encouraging him to go on with life: “It was really meaningful. Through this experience, he will be able to cope for another day.” (GN17). 

One musician pointed out that, in addition to the musical experience itself, the per-former’s approach can be a meaningful catalyst for new ways of caring and communication, creating new communities and transforming people’s lives in the hospital environment: “In fact, the musicians and the music can bring something new. It can open up life [for new perspectives] to come and enable people to start talking to each other and commenting a little, and you [the nurse] get a little change from everyday triviality.” (GM3). Another described the treatment room as an intimate space where the musician was close to the patients and, e.g., felt a very close and present contact with a male patient, as if both were sharing the musical moment and performing together: “When patients spontaneously said, “It’s nice to have something positive to think about” and “It’s a great atmosphere you can create with your guitar,” it brought a feeling of togetherness into the room.” (GM1).

When the patients’ behavior suggested detachment, e.g., eyes closed or watching television, this made the task more complex for the musicians (RJ4). However, as the musicians performed repeatedly for the same group of patients over the project period, better mutual contact developed between some of these patients and the musicians (RJ1). At one of the final music interventions, a patient requested his favorite song (IP7), and the patients in the room sang along together with the musician. The musician noted that this was a very special and life-affirming moment.

For patients and nurses, the music was a shared experience that gave rise to feelings of joy, cohesion, openness, and community. It was a happy experience that they could talk about afterwards: “So what I experience is that the music creates calmness, cohesion, and community. And that is something we can use to build upon. For all the other times [HD treatments]. And that’s what I’ve been thinking here: that it’s really nice.” (IN1). The nurses emphasized that the music made a positive difference to their workday and that there was a positive and expectant mood among patients and staff in the morning before the musicians arrived. The musicians noted that, when they entered the treatment rooms, they also felt that the patients were looking forward to the music. One patient commented: “It´s nice to be together with people who express joy in music.” (IP7). In addition, the music may balance and dissolve the divisions between the roles of patients and staff, thereby creating a new and deeper mutual understanding. This point was supported by a nurse: “We work in a unit where it is necessary that you can also show yourself as a person to the patients.” (IN1).

#### 3.5.2. Subtheme 2b: The Artistic Quality Mediating a Magnificent and Beautiful Experience 

Many nurses and patients described the musicians’ presence, talent, personality, and approach as central to their experience of and engagement with the intervention. The nurses were impressed by the musicians, their professionalism, and their way of being present and adaptive in the treatment rooms: “It´s a great gift and an opportunity to have real musicians here who play live music, and who perform so nicely and beautifully.” (IN5). One noted that the musicians were very attentive and humble, unaffected by the alarms of the machines: “They are extremely attentive. Really. Probably also because they are so musical and therefore creative and very sensitive people.” (IN10). The patients were also impressed by the musicians´ artistic level, and some mentioned that seeing how the musicians performed was a positive and reflective experience: “It’s nice when you look at the musicians, how they are active and such. And it gives you something. Then you can follow what they do and such. There is a lot of hard work behind it.” (IP11).

The musicians experienced the staff as friendly, accommodating, and supportive: “The staff at the HD department were very welcoming and committed, and I think it was a very professional and a satisfying experience.” (GM1). The nurses’ commitment to being open-minded and engaged with the live music performances in the treatment rooms had a huge impact on the musicians. They felt that the nurses’ professional approach created a safe and stable environment, which helped in situations, for instance, when patients lay with eyes closed, making them difficult to read (GM2). Sometimes, the nurses gave the musicians appreciative looks and commented on the music, and it seemed that the musicians saw the nurses both as a link to the patients and as part of the audience in the same way as the patients: “But there was something that I thought was really cool. Not just to play for the patients, but also to play for you [nurses] who were in the treatment rooms.” (GM3).

Meeting patients on their own terms and adapting to their state of mind required a sensitive, intuitive, and empathetic approach. While the musicians’ attention was directed outward, they had to accentuate the calm nuances of the music rather than “filling the space” as they would in the familiar environment of a concert hall. The musicians matched the intensity of the music to the state of mind and energy levels of the patients in the treatment rooms; therefore, they sometimes changed the tempo or even the order of the repertoire according to the patients’ feedback. The musicians created a safe and positive space, inviting the patients to participate by asking questions, commenting, saying what they experienced during the music, and bringing memories to life. One musician described it in the following way: “That way, the patient becomes more active and is invited to join in and as a natural consequence I think that the patient will listen more deeply and differently to what is played on the guitar.” (GM1). If the patients were passively listening, for instance with eyes closed, the musicians noted that they would play more tenderly, dreamily, and quietly. In contrast, they would choose to play accessible, energetic, and upbeat music for attentive patients who were seeking contact. One musician felt that this intuitive and adaptive approach had implications for the musicians’ professional and personal development: “Participating in this space is like a development springboard both as a musician and as a human being.” (GM3). Experiencing the diversity of the patients’ respective personalities and reactions in this intimate space meant the musicians had to be open, artistically aware, and prepared for the unpredictable.

## 4. Discussion

Our data show that patient-tailored live music performed by professional musicians creates both an inner space and a participating space—a musical break. This musical break, alone or with others, represents a respite from the HD treatment—a temporary diversion from the burdensome treatment and stressful work environment, allowing patients’ and nurses’ thoughts to run free and create new perspectives. We found that patient-tailored live music interventions fostered well-being among patients undergoing HD, improved patient–nurse relationships and relaxation among nurses, and created a new artistic and social awareness for the professional musicians entering this healthcare environment. The themes identified showed that the music interventions were a meaningful break that influenced mental and physical well-being, time perception, community, professional attitudes, and artistic approach. The artistic quality of the music was essential, together with the musicians’ social awareness, empathy, and ability to interact with the patients, in creating meaningful moments for patients and staff. Overall, the music interventions were a welcome and positive change in a predictable world of stressful routines and repetitive treatments.

For some, the music created a mutual consciousness, sensitivity, and attunement between patients, nurses, and musicians. It seemed that a third common space arose—a “we”—a resonant space of reaching out and letting go of negative thoughts, regardless of one’s role as a patient or nurse. It is possible that the music created an autonomous space—an “intermediate world” away from the medical hierarchy—as well as a sound environment, where patients, staff, and musicians could connect and communicate, without words, through music [40]. According to the philosopher Jørgensen, the inter-mediate world is characterized by openness and being present in the world in its most original state: a place where we can find the source of imagination, experience, and cognition that open minds, allowing possibilities to become perceptible [41]. Furthermore, the intermediate world “is the sensitive subjectivity in which experience of transcendence occurs before any crystallization of subject and object has taken place”—a place outside time and place [41]. We found that the music interventions took the patients, nurses, and musicians out of their current time and space, enabling them to catch the moment, opening minds, and allowing new thoughts to arise on their own terms, individually or as a group.

Our findings are in line with Heidegger’s theories that being in the world is a predictor for being within the world and the existence of an understanding of the presence of others [39]. Heidegger also dissolves the distinction between subject and object. For many patients on HD, treatment is lifelong, and therefore building up mutual communities and a meaningful environment would be helpful to both patients and nurses [8,24]. It is possible that live music may help dissolve the barriers between the roles of patients and nurses in the HD setting. This effect was seen in a surgery ward where participatory music making with healthcare professionals and professional musicians led to a more balanced hierarchy in the ward, with this musical approach acting as an agent of change in participants becoming more mindful of the present moment [23]. Our results show that live music has the potential to counteract feelings of meaninglessness and wasted time, as found in Gullick et al. [8]. Live music may also contribute to humanizing the HD environment by creating a feeling of “being-with” and present with others. This is the opposite effect to the pre-recorded music applied in most HD music intervention studies, which creates individual musical zones and cannot respond to patients’ reactions during the intervention.

The strategy of combining well-known music with unfamiliar music aimed to pique the patients’ curiosity and also open them up to new perspectives on the music they already knew. The music intervention created an inner space where patients could experience memories from their past. Throughout life, many people build up an individual soundtrack—a musical memory lane—consisting of specific genres or pieces of music related to meaningful life events [42,43,44,45]. According to North and Hargreaves, music played during childhood in particular fosters a sense of cohesion and fellowship and becomes ”the musical story of my life” by adding an emotional anchoring of “who I am” when alone and with others [46]. Data from our study show that the musicians in many cases managed to activate these individual soundtracks among the patients, bringing up a positive memorable moment from the past and creating a positive atmosphere in the ward, possibly improving the quality of life and counteracting existential feelings of boredom, as mentioned in Moran et al. [7].

According to Ruud, we construct ourselves when we listen to music [43]. Music evokes many different feelings, from relaxation to excitement, happiness to sadness, fear to safety, or even a combination of these [47]. While previous evidence suggests that this musical self-contraction might be a resource for patients whose self-perception of identity is challenged, for instance, when they are diagnosed with cancer [40,48,49], our data suggest that this might also be a resource for patients on HD who listen to live music during treatment. Art-based interventions for patients on HD have also been found to improve self-esteem and motivation, increase social interaction, and improve the overall HD experience [50].

This self-construct might also counteract feelings of burnout among nurses in the HD setting, on both a personal and professional level. It is potentially a way to manage a stressful work environment, as well as creating shared experiences and pathways of communication with the patients. Our results are in line with Carswell et al., showing that individual art-based interventions improve well-being among nurses working in an HD unit [10,50].

Another key finding is that patients and nurses felt that the artistic quality of the music was important to the experience. These findings resemble those of Smilde, arguing that “authenticity of sound,” “fitness for purpose,” and “relevance to context” are important elements in achieving quality, and therefore meaningfulness, when involving music in healthcare contexts [51]. In this study, professional musicians were guided to react to the different responses from the patients and create bonds through an emotional response, in contrast to two other live music interventions in an HD setting where nurses performed for the patients [9,10]. To succeed, healthcare musicians need to focus on the aspects outlined in Heidegger’s framework: authenticity, commitment, taking risks, and embracing a specific kind of possibility for being in the world [39]. Our study identified an artistic quality together with the musicians’ social awareness, empathy, and intuition as central to and prerequisites for the patients and staff being able to immerse themselves in the musical experience.

### Strengths and Limitations

This study was part of a randomized controlled pilot trial and aimed to garner an in-depth understanding of the patient, staff, and musician perspectives. One limitation of this study may be that the primary investigator had the roles of both selecting and supervising the musicians, as well as analyzing the data, including the reflective journals. This might have influenced the perspectives and reflections on the data. On the other hand, a strength of the analysis was that the second and last co-authors also participated in the in-depth analysis and brought different perspectives and reflections to the analysis and discussion of the findings. The study took place on a single site; thus, multiple sites might discover further perspectives. Another limitation could be that the positive feedback from the patients and nurses might be explained by novelty bias, why implementing patient-tailored live music into clinical practice maybe will show some other findings.

A major strength of this study was the overall sample size with 18 moderate participation observations, 17 semi-structured interviews with patients, 9 semi-structured interviews with nurses, 1 semi-structured group interview, and 13 reflective journals from the musicians. These provide strong data material seen from a phenomenological-hermeneutical perspective. Furthermore, the various methods used to gather data are also a strength when bringing forth various perspectives to the phenomenon “patient-tailored live music intervention” [33,34].

## 5. Conclusions

We found that patient-tailored live music performed by professional musicians had positive effects on mental and physical well-being, time perception, and community among patients undergoing the burdensome treatment of HD. For the nurses, the music was relaxing and de-stressing, and had a positive effect on their work by improving their patient relationships and compassion. For the professional musicians, their artistic and social awareness was enhanced when they entered the HD setting. The artistic quality of the music was essential—together with the musicians’ social awareness, empathy, and ability to interact with the patients—in creating meaningful moments for the patients and staff.

## Figures and Tables

**Table 1:** Overview of data collection.

Aspect	Method	During Intervention	Post-Intervention	One Month after the Intervention Period
The non-verbal environment	Moderate participation observations (patients/nurses/musicians)	X		
Personal experience (well-being, meaningfulness)	Semi-structured interviews (patients)		X	
Personal and professional experience	Semi-structured interviews (nurses)		X	
Interprofessional cooperation	Semi-structured group interview (nurses/musicians)			X
Artistic and social awareness	Reflective journals (musicians)		X	

**Table 2:** Study participant characteristics.

	Patients (N = 12)	Nurses (N = 17)	Musicians (N = 4)
**Characteristics**	Mean (SD) (min/max) or numbers (%)		
Age	65 (20.1) (24–84)	55 (10.3) (28–66)	37 (16.5) (21–60)
Female	6 (50%)	16 (94%)	1 (25%)
Months on HD	39.7 (24.5) (9–87)	-	-
Years working in HD unit	-	13.3 (9.0) (1–23)	-
Years working as professional musician	-	-	16.5 (16.4) (2–40)
Cohabiting	7 (58%)	-	-
Unemployed	12 (100%)	-	-

**Table 3:** Themes, subthemes, and quotes illustrating meaningfulness.

Theme	Subtheme	Example quote
1. The inner space	(1a) Entering a calm and enjoyable pause bubble	“I’m kind of a little more relaxed in my body. I just fall down a little bit more.” (IP6) “Really soothing.” (IP10) “It was just so beautiful. So ... Then the thoughts come a little that way, yes.” (IP1) “A pause bubble ... It was just getting out of stress, and just getting down and breathing.” (IN10) “Music is all about letting go—letting go of worries and bad energy.” (IN2)
	(1b) Resting in a thought-free state of mind	“I was entering my own space.” (IP1) “It’s nice that it [experiencing the music] makes time go faster, in a way.” (IP5) “Your heart rate goes all the way down and you just get your head emptied and that gives such a break.” (IN10)
	(1c) Traveling in the past and catching the moment through heartfelt music	“Like traveling in the past.” (IP1) “When it was so quiet and so calm, I thought of the sea. The waves that came rushing just as quietly and so calm, that is. You can imagine some pictures.” (IP11) “The music had to be chosen and played with your heart.” (RJ1)
2. The participating space	(2a) Bringing positive changes into life	“It´s nice to be together with people who express joy in music.” (IP7) “It was really meaningful. Through this experience, he [the patient] will be able to cope for another day.” (GN17) “We work in a department where it is necessary that you can also show yourself as a person to the patients.” (GN1) “Music creates cohesion and community. And that is something we can use to build upon. For all the other times [HD treatments].” (IN1) “It can open up life [for new perspectives] to come and enable people to start talking to each other and commenting a little, and you [the nurse] get a little change from everyday triviality.” (GM3)
	(2b) The artistic quality mediating a magnificent and beautiful experience	“It´s a great offer and an opportunity to have real musicians here who play live music, and who perform so nicely and beautifully.” (IP5) “It’s nice when you look at the musicians, how they are active and such. And it gives you something.” (IP11) “Participating in this space is like a development springboard both as a musician and as a human being.” (GM3) “The nurses created a lovely space to visit, and I was surprised by their support without showing their stressful workday.” (RJ2)

## Data Availability

The dataset for this qualitative study can be accessed by contacting the authors of the paper.
